# Improvement of endocytoscopic findings after per oral endoscopic myotomy (POEM) in esophageal achalasia; does POEM reduce the risk of developing esophageal carcinoma? Per oral endoscopic myotomy, endocytoscopy and carcinogenesis

**DOI:** 10.1186/1471-230X-13-22

**Published:** 2013-01-30

**Authors:** Hitomi Minami, Naoyuki Yamaguchi, Kayoko Matsushima, Yuko Akazawa, Ken Ohnita, Fuminao Takeshima, Toshiyuki Nakayama, Tomayoshi Hayashi, Haruhiro Inoue, Kazuhiko Nakao, Hajime Isomoto

**Affiliations:** 1 Department of Gastroenterology and Hepatology, Nagasaki University Hospital, 1-7-1, Sakamoto, Nagasaki 852-8501, Japan; 2 Department of Pathology, Nagasaki University Hospital, 1-7-1, Sakamoto, Nagasaki, 852-8501, Japan; 3 Digestive Disease Center, Showa University Northern Yokohama Hospital, Yokohama, Japan

**Keywords:** Esophageal achalasia, POEM, Per-oral endoscopic myotomy, Esophageal cancer, Squamous cell carcinoma

## Abstract

**Background:**

Per oral endoscopic myotomy (POEM) has been reported to be a new therapeutic option for esophageal achalasia. The possibility that POEM could reduce the risk of developing esophageal squamous cell carcinoma was evaluated.

**Methods:**

This was a single-centre, retrospective study. Fifteen consecutive patients with esophageal achalasia who underwent POEM in our institution between August 2010 and January 2012 were enrolled. Ultra-high magnification with endocytoscopy was performed, and both histopathological and immunohistochemical evaluations for Ki-67 and p53 were assessed before and 3 months after POEM.

**Results:**

POEM was successfully performed and effectively released the dysphagia symptom in all patients without severe complications. Subjective symptoms (mean Ekcardt score, before 7.4 vs. after 0.5, p<0.05) and manometric pressure studies (mean lower esophageal sphincter pressure), before 82.7 vs. after 22.9 mmHg, p<0.05) showed substantial improvement following POEM. The average numbers of esophageal epithelial nuclei before and after POEM on endocytoscopic images were 128.0 and 78.0, respectively (p<0.05). The mean Ki-67-positive ratio was 26.0 (median 25.4, range, 10.3-33.2) before and 20.7 (median 20.0, 13.1-29.9; p=0.07) after POEM, and the mean p53-positive ratio was 2.35 (median 2.61, 0.32-4.23) before and 0.97 (median 1.49, 0.32-1.56; p<0.05) after POEM. A significant positive correlation was seen between the number of nuclei and the Ki-67-positive ratio (p<0.05).

**Conclusions:**

POEM appears to be an effective and less invasive treatment of choice against achalasia and may reduce the risk of esophageal carcinogenesis. Endocytoscopy can be useful for the assessment of esophageal cellular proliferation.

## Background

Achalasia is a rare, chronic, esophageal motility disorder with an estimated annual prevalence of 1 per 100,000 subjects in Western populations [1]. The disease can occur at all ages, but the incidence seems to increase with age. The predominant symptoms are dysphagia and regurgitation, because of the impaired relaxation of the lower esophageal sphincter (LES) and the loss of normal peristalsis [2]. Persistent esophageal distension with retention of foods and fluids, bacterial overgrowth, and impaired clearance of regurgitated acid and gastric contents lead to chronic inflammation and passively cause dysplasia and carcinoma [3-5]. The aim of therapy is to reduce food stasis, which might interrupt the progression to carcinoma and thus reduce the risk of squamous cell carcinoma. In 1872, Fagge reported the first case of esophageal carcinoma developing in a patient with achalasia [6]. The reported incidence of concomitant carcinoma and achalasia ranges from 0.53% to 8.6% [7]. In a recent study, Meijssen et al. reported that the risk of developing squamous cell carcinoma in achalasia patients was increased 33-fold over that in the general population [8]. Leeuwenburgh et al. also reported that the relative hazard ratio of esophageal cancer was 28 (confidence interval 17–46) compared with an age- and sex-identical population in the same timeframe [1]. Although many investigators have warned clinicians about the high incidence of carcinogenesis in achalasia [9], the prognosis of patients who develop such carcinomas remains poor.

Recently, per oral endoscopic myotomy (POEM) was introduced by Inoue et al. [10] as a less invasive and effective curative treatment option with minimal complications. In the present study, the clinical feasibility of POEM in achalasia patients in our hospital was evaluated. In addition, whether this innovative procedure could affect esophageal epithelial proliferation was examined by ultra-high magnification with endocytoscopy, along with immunohistochemical staining of targeted biopsies with antibody against Ki-67 nuclear antigen before and after POEM. Immunohistochemistry with anti-p53 antibody was also used to assess the alteration in squamous cell carcinoma risk with POEM.

## Methods

### Design

This was a single-centre, retrospective study. This study was approved by ethics committee of Nagasaki University Hospital.

### Patients

Fifteen consecutive cases of esophageal achalasia which underwent POEM in our institution between August 2010 and January 2012 were enrolled in this study. A clinical summary of the 15 patients (3 males, 12 females; mean age 51.7 years, age range 26–84 years) with esophageal achalasia is shown in Table 1. The dilation grade of the disease was II in 6 patients and III in 1 patient. Prior endoscopic balloon dilation was performed in 5 patients. Two patients with sigmoid type achalasia were included.

**Table 1:** Patients’ characteristics

**No.**	**Sex**	**Age (years)**	**BMI (kg/m2)**	**Dilation**	**Type**	**Balloon dilation**	**Systemic complication**
1	female	60	20.3	II	straight	-	none
2	female	50	17.1	II	straight	-	none
3	male	54	21.9	III	sigmoid	3 sessions	hypertension
4	female	63	18.4	II	sigmoid	-	breast cancer
5	female	45	23.2	I	straight	-	rheumatoid arthritis
6	female	26	18.0	I	straight	-	none
7	female	38	18.5	I	straight	-	none
8	female	47	22.4	II	straight	1 session	none
9	female	84	26.5	I	straight	-	hypertension
10	female	79	18.7	I	straight	1 session	pulmonary emphysema
11	male	43	22.4	I	straight	-	none
12	male	69	17.0	I	straight	5 sessions	pulmonary emphysema
13	female	46	19.0	I	straight	2 sessions	none
14	female	37	21.8	II	straight	-	none
15	female	38	25.4	II	straight	-	none
maen		51.7	20.7				

Written informed consent was obtained from all subjects, a legal surrogate, the parents or legal guardians for minor subjects prior to their inclusion, in accordance with the Helsinki Declaration and under approval by the Nagasaki University Ethics Committee. All the subjects also consented for the publication of individual clinical details with a written informed consent.

### Dysphagia symptom scores

To evaluate improvement of dysphagia objectively, a dysphagia symptom score and the Eckardt score (representative subjective symptom score) were used [11]. The dysphagia symptom score was defined as follows: the worst degree of dysphagia just before the treatment was scored as 10, and the best condition, before the patient developed achalasia, was scored as zero, in accordance with Inoue’s report [10]. The post-procedural degree of dysphagia was scored between 0 and 10 in each patient. Scoring was done before POEM, after POEM on the day of discharge from hospital, and 3, 6, and 12 months later.

### Barium esophagogram

The degree and shape of achalasia were assessed by barium passage. The maximum diameter of the thoracic esophagus was measured, and achalasia was classified into grade I (diameter <3.5 cm), grade II (3.5 cm < diameter < 6.0 cm), and grade III (diameter ≥6.0 cm), in accordance with the descriptive rules (Descriptive Rules for Achalasia of the Esophagus, Japan Society of Esophageal Diseases, June 2012, 4th Edition). On the day following POEM, a barium swallow was done to confirm passage of contrast media through the gastroesophageal junction (GEJ) without leakage. Achalasia shapes include straight, flask, and sigmoid types.

### Esophageal manometry

Esophageal pull-through manometry was done in all cases before and after the procedure using the Polygraf ID (Medtronic Functional Diagnostics, Denmark). According to the manufacturer’s data, the normal range for LES pressure with this device is from 14.3 to 34.5 mmHg. The manometry study could not be completed in case 15 before POEM because the measuring catheter did not pass through the narrow segment of the esophagus.

### POEM

The POEM procedure received approval from the institutional review board (IRB) of Nagasaki University Hospital (approval number 10052824, issued on May, 22 2010). Written informed consent was obtained from all patients.

A forward-viewing endoscope with an outer diameter of 9.8 mm (GIF-H260; Olympus, Tokyo, Japan), which is routinely used for upper gastrointestinal examination, was used with a transparent distal cap attachment (MH-588, Olympus). A triangle-tip knife (KD- 640 L; Olympus) was used to dissect the submucosal layer and to divide circular muscle bundles. A coagulating forceps (Coagrasper, FD-411QR; Olympus) was used to coagulate larger vessels prior to dissection and for hemostasis. The VIO 300D (ERBE, Tubingen, Germany) electrogenerator was used, and for final closure of the mucosal entry site, hemostatic clips (EZ-CLIP, HX- 110QR; Olympus) were applied. The procedures were performed under general anesthesia with positive pressure ventilation. The UCR CO_2_ insufflator (Olympus) was used with a regular insufflating tube (MAJ-1742; Olympus), which maintained CO_2_ insufflation at a constant rate of 1.2 L/min.

**Figure 1:**
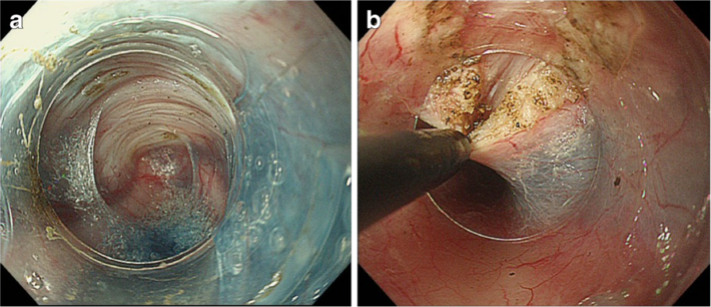
**POEM procedures.** 1-**a**. A long submucosal tunnel is created. The blue color of the indigocarmine that was mixed with the glycerin solution helps discriminate the white muscle layer from submucosal tissue.1-**b**. Using a triangle-tip knife, the inner circular muscle is hooked and cut. Outer longitudinal muscles are seen behind the inner muscles. Outer muscles should be preserved to avoid severe complications such as compartment syndrome and mediastinitis.

The POEM procedures were done as previously described with slight modifications as follows. Submucosal injection was done first at the level of the middle thoracic esophagus, approximately 13 cm proximal to the GEJ. About 10 mL of saline supplemented with 0.3% indigocarmine were injected. A 2-cm, longitudinal mucosal incision was made on the mucosal surface to create a mucosal entry to the submucosal space (dry cut mode, 50 W, effect 3). Then, a technique similar to endoscopic submucosal dissection (ESD) was used to create a submucosal tunnel downwards, passing through the GEJ, and about 3 cm into the proximal stomach (Figure 1a). Spray coagulation mode (60 W, effect 2) was used to dissect the submucosal layer. Larger vessels in the submucosa were coagulated using the forceps in the soft coagulation mode (80 W, effect 5). Dissection of the circular muscle bundle was started at 2 cm distal from the mucosal entry. The sharp tip of the knife was used to capture only circular muscle bundles and then lift up toward the tunnel lumen (Figure 1b). The captured circular muscle bundle was cut by spray coagulation current (60 W, effect 2). Division of the sphincter muscle was continued from the proximal side towards the stomach until it passed 2 or 3 cm distal from the LES. After completion of the myotomy, smooth passage of the endoscope through the GEJ with minimal resistance was confirmed. The incised mucosal entry was closed with about five hemostatic clips after prophylactic antibiotic solution was sprayed inside the tunnel. Successful closure of the mucosal entry was confirmed by the endoscopic appearance. At the end of the procedure, the endoscope was again inserted into the original esophageal lumen down to the stomach, to confirm smooth passage through the GEJ.

### Endocytoscopy

**Figure 2:**
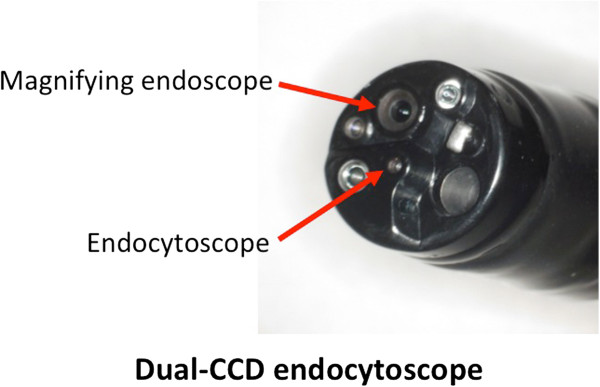
**The dual-CCD endocytoscope carries lenses for both conventional magnification and ultra-high magnification.** This scope enables us to obtain ultra-high magnifying images during usual endoscopic examination.

A prototype endocytoscope with an integrated dual charge-coupled device (CCD) (Figure 2) (GIF-Y0001, Olympus) was used. The dual-CCD integrated type endoscope has both conventional magnification (80-fold) and ultra-high magnification (450-fold) capabilities, which can be easily interchanged at the push of a button on the endoscope. The clinical use of the prototype endoscope was approved by the hospital’s ethics committee (IRB approval number 10102236, Oct. 29 2010), and written informed consent was obtained from all patients. A mixture of 0.05% crystal violet and 0.1% methylene blue was used during endocytoscopy to obtain images similar to conventional pathological images using hematoxylin and eosin staining. Crystal violet effectively dyes the cytoplasm, whereas methylene blue allows detailed identification of the cell structure, including the nuclei and cytoplasm. Dilution of methylene blue with crystal violet seems to improve visualization of the lesion and may also decrease the potential risk of DNA damage due to a higher concentration of methylene blue [12]. Patients underwent both conventional endoscopy and endocytoscopy at the same time under conscious sedation with intravenous pethidine hydrochloride (35 mg; Opystan, Mitsubishi Tanabe Pharma, Osaka, Japan), supplemented with diazepam (5–10 mg, Takeda Pharmaceutical, Osaka, Japan). After conventional endoscopic observation, ultra-high magnification was applied. The mucosa on the posterior wall 25 cm distal from the incisor was targeted. Still images were obtained from the targeted area, and 3 images of sufficient quality were selected for evaluation. Then, the average nuclei counts of the 3 images before and 3 months after POEM were compared. After endocytoscopic observation, biopsies were taken from the targeted area. All endocytoscopy examinations were recorded, and the location photographed during endocytoscopy coincided with the histopathological images. Nevertheless, complete correspondence between endocytoscopic images and microscopic images is still limited [13].

### Immunohistochemistry using anti-Ki-67 and anti-p53 antibodies

Four-mm tissue sections of the formalin-fixed, paraffin wax-embedded samples were sliced and mounted on silane-coated glass slides, dried, deparaffinized with xylene and rehydrated through graded ethanol. Antigen retrieval was performed by boiling for 15 minutes in 10 mM monocitric acid buffer (pH 6.0) for anti-Ki-67 staining and in 10 mM Tris/ethylenediaminetetraacetic acid buffer (pH 9.0) for anti-p53 staining. Prior to immune staining, endogenous peroxidase activity was blocked by incubating the slides in a 0.5% solution of H_2_O_2_ in phosphate-buffered citric acid for 15 minutes at room temperature. After washing 3 times with Tris-buffered saline (TBS) with a pH of 7.4, the sections were incubated in TBS buffer containing 10% rabbit non-immune serum (DAKO, Glostrup, Denmark) and 10% normal human plasma (and 5% bovine serum albumin [BSA] for p53 staining) for 20 minutes. Then, the sections were incubated with the primary antibody against anti-human Ki-67 antigen (clone MIB-1, DAKO) in a 1:100 dilution or p53 antigen (clone DO-7, DAKO) in a 1:100 dilution for 14 hours at 4°C. Subsequently, biotin-labeled rabbit-anti-mouse antibody (DAKO) was used as the second antibody, followed by the addition of a streptavidin-horseradish peroxidase complex (DAKO). To detect Ki-67 and p53, 3-amino-9-ethylcarbazole was used as the substrate. One experienced pathologist who was blinded to the clinical data counted at least 300 nuclei in each sample. Cells were counted as positive when moderate to intense nuclear staining was found. Staining with the isotype immunoglobulin and diluted solution was performed in the same fashion for the negative controls.

### Statistics

Statistical analyses were performed using Fisher’s exact test, the χ^2^ test, Student’s *t*-test, the Mann–Whitney U test, and the Kruskal-Wallis test, as appropriate. A P value <0.05 was accepted as significant. Data are expressed as means ± standard deviation (SD).

## Results

### POEM

POEM was successfully performed in all 15 cases without severe complications such as perforation and mediastinitis. Details of the procedure outcomes are shown in Table 2. No massive pneumoperitoneum that required reduction of abdominal distention or capnomediastinum was observed. Two patients, who had a long disease history and severe submucosal fibrosis due to preceding repeat balloon dilation, experienced post-procedural bleeding, which resolved with conservative management. The mean LES pressure decreased from 82.7 to 22.9 mmHg, and the subjective symptom scores were also significantly lower after POEM (Ekcardt score p < 0.05, dysphagia symptom score p < 0.05).

**Table 2:** Outcomes of the POEM procedure

**No**	**Procedure time (min)**	**Symptom score**	**LESP(mmHg)**		**Eckardt score**		**Myotomy length**	**Complications**
			**Before**	**After**	**Before**	**After**		
1	98	0	70.0	16.4	3	0	14	none
2	160	1	87.0	35.0	8	1	15	none
3	140	1	50.4	28.2	3	0	15	bleedind(day 10)
4	120	0	48.0	37.4	8	0	13	none
5	80	3	98.0	33.5	7	1	12	none
6	100	0	109.5	25.1	9	0	15	none
7	88	3	56.5	23.6	7	3	18	none
8	90	0	104.1	15.2	9	0	14	none
9	85	0	119.0	28.0	5	0	15	none
10	75	3	256.0	26.7	6	1	10	none
11	80	0	65.8	14.7	9	1	18	none
12	140	1	73.0	29.6	8	1	18	bleedind(day 1)
13	90	2	29.8	19.5	4	1	15	none
14	75	0	46.7	6.7	6	0	17	none
15	95	0	-	14.7	12	0	14	none
mean	101.1	0.7	82.7	22.9	7.4	0.5	14.9	

Even after a mean follow-up of 11.3 (range 8 – 25) months, no patient developed recurrent symptoms of dysphagia. Endoscopic reflux esophagitis was identified in 5 patients (33.3%) on follow-up endoscopy examination. Two (15.3%) patients received proton pump inhibitor (PPI) therapy, and the symptoms were easily controlled.

**Figure 3:**
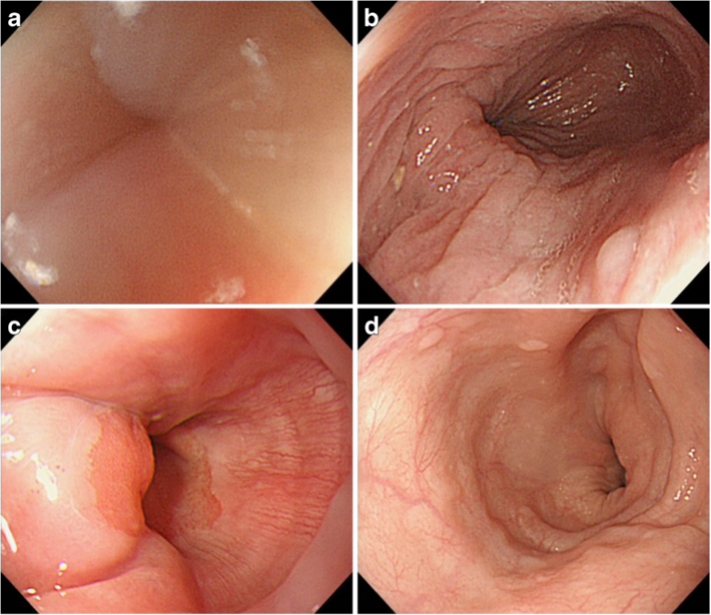
**Conventional endoscopic images of the EGJ and mid-thoracic esophagus before and after POEM.** 3-**a**, **b** Before POEM, the EGJ is extremely tight. Whitish color change is seen on the surface mucosa without permeability of vascular structures. 3-**c**, **d** After POEM, the narrow segment of the EGJ has been totally released. The status of the surface mucosa is significantly improved, and permeability of the surface vascular patterns is restored.

Figure 3a-d shows the endoscopic images taken from the GEJ and mid-thoracic esophagus before and after POEM. Before POEM, the endoscope was passed through the GEJ with great resistance. The thickened esophageal mucosa showed turbidity and whitish color change, lacking permeability of the vascular structures. Following POEM, however, the resistance to endoscopic passage through the GEJ was completely resolved, and the mucosal abnormalities were substantially improved, without the whitish color change. The POEM procedure allowed detailed observation of the vascular structures of the mucosal surface.

### Endocytoscopic findings

Thirteen achalasia patients underwent endocytoscopy and targeted biopsy before and after POEM. In Case 1, informed consent for the study was not obtained before the procedure, and Case 6 was excluded due to concerns about possible adverse effects of methylene blue on her pregnancy.

**Figure 4:**
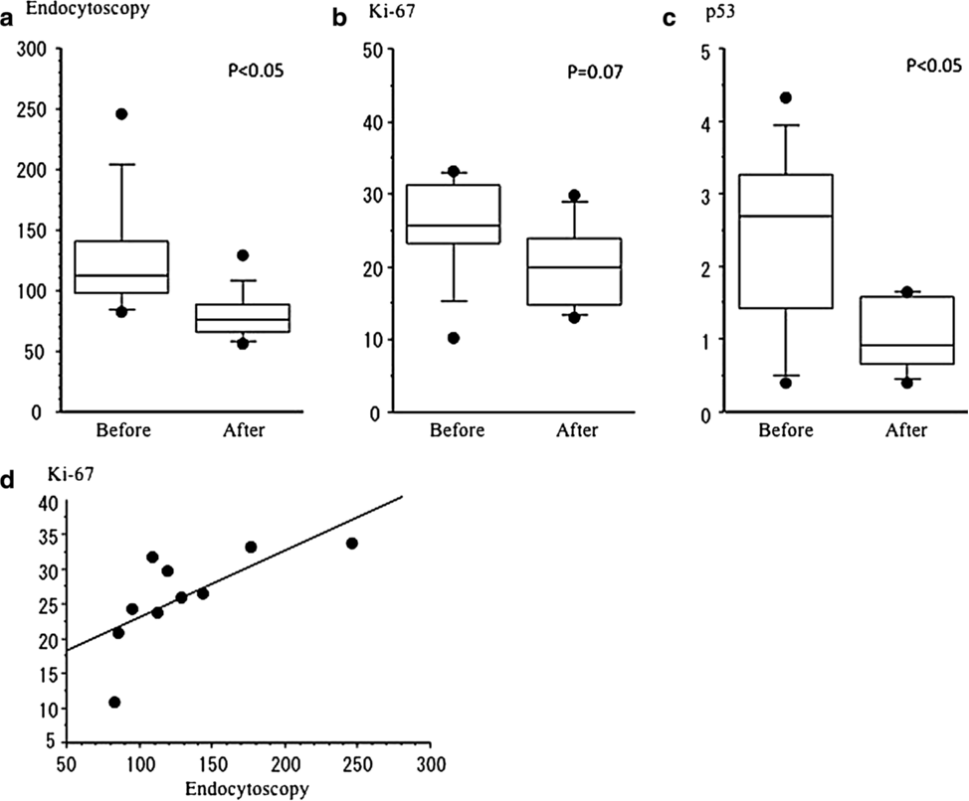
**a-d. The endocytoscopic counts of nuclei are significantly improved after POEM (a).** Also, the mean Ki-67-positive ratios before and after the procedure are 26.0 (median 25.4, 10.3-33.2) and 20.7 (median 20.0, 13.1-29.9), respectively (p = 0.07) (**b**, **c**). The mean p53-positive ratios before and after the procedure are 2.35 (median 2.61, 0.32-4.23) and 0.97 (median 1.49, 0.32-1.56). There was a significant positive correlation between the esophageal epithelial nuclei numbers assessed by endocytoscopy and the Ki-67-positive ratios (correlation coefficient, 0.695, p < 0.05) (Figure 4**d**).

**Figure 5:**
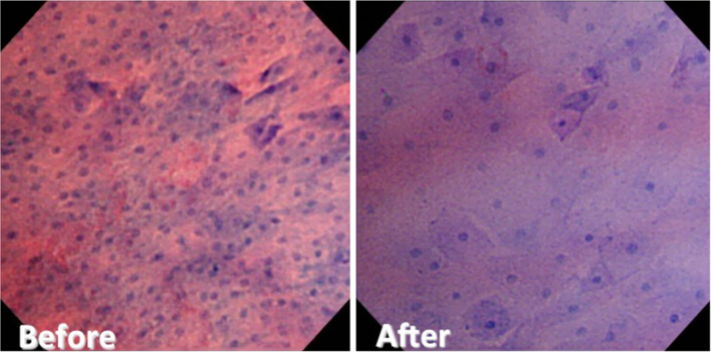
**Comparison of endocytoscopic images between before and after POEM, including the shape and size of cells and the number of nuclei.** The number of surface cells and cellular arrangement was significantly improved after POEM.

The average counts of esophageal mucosal nuclei on endocytoscopy before and 3 months after POEM were 128.0 (median 112.3, range 82.7-246.0) and 78.7 (median 76.0, 56.7-129.3) (Figure 4a), respectively. Thus, POEM significantly reduced the number of nuclei (p < 0.005). Compared to the endocytoscopic images obtained before POEM, cellular and nuclear structures and inter-cellular margins were observed more clearly in the images obtained after POEM (Figure 5). The nuclei numbers before POEM varied very widely, whereas they were distributed in a relatively narrow range after the procedure.

There were no significant correlation between endoscopic nuclear counts and patients’ characteristics such as degree of dilation, type, and duration of dysphagia symptom. However, statistically significant correlation between endocytoscopic nuclear counts and Eckardt score before the procedure was observed (r = 0.7937, 0.3699 ~ 0.9441, p < 0.01).

The number of nuclei in 3 non-achalasia patients (healthy controls) was also evaluated, and the average count was 88.5 (range, 76–97), which was the same level as the post-POEM values in achalasia patients.

### Immunohistochemical analysis: Ki-67 and p53 immunostaining

The mean Ki-67-positive ratios before and after the procedure were 26.0 (median 25.4, 10.3-33.2) and 20.7 (median 20.0, 13.1-29.9; p = 0.07), respectively (Figure 4b). There was a significant positive correlation between the esophageal epithelial nuclei numbers assessed by endocytoscopy and the Ki-67-positive ratios (correlation coefficient, 0.695, p < 0.05) (Figure 4d).

The p53-positive ratios were decreased in the post-treatment group compared to the pre-treatment group with statistical significance. The mean positive ratios before and after the procedure were 2.35 (median 2.61, 0.32-4.23) and 0.97 (median 1.49, 0.32-1.56), respectively (Figure 4c). Histopathological evaluation with hematoxylin and eosin staining confirmed that all of the specimens were negative for dysplasia or squamous cell carcinoma.

## Discussion

Recently, POEM has been developed in Japan [10] as a less invasive and effective curative treatment option with no cases of severe complications. Other therapeutic options include endoscopic balloon dilatation and surgical myotomy. Endoscopic balloon dilatation is still widely performed because of its relative noninvasiveness and simplicity, but it has a relatively lower success rate and often requires multiple treatment sessions [14]. Inoue et al. reported that there was no recurrence after the POEM procedure in their 17 case series of achalasia in their short-term results [10]. They also reported the lack of recurrence on 43 patients of maximum of 1 year 9 months observation [15] (Japanese manuscript). In the present 15 cases, no severe complications such as perforation and severe mediastinitis occurred, and the clinical course was uneventful in each case. Although the term has been relatively short, there has been no disease recurrence during the observation period. Surgical myotomy has been thought to be the curative therapeutic choice for symptomatic achalasia. However, it requires skin incisions and additional anti-reflux surgical intervention. Thus, POEM could be one of the most promising treatment options for symptomatic achalasia.

On the basis of prior data, the overall prevalence of esophageal squamous cell carcinoma in patients with achalasia has been estimated to be up to 8.6%, accounting for a 50-fold increase in cancer risk [7]. In achalasia patients, it has been suggested that chronic food stasis leads to chronic inflammation, epithelial hyperplasia, multifocal dysplasia, and squamous cell carcinoma (SCC) [1,16,17]. Previous studies showed that it would take a mean of 24 (range 10–43) years to establish carcinoma after symptom onset, and 3 to 4 years for carcinoma to develop from dysplasia [1,18,19]. Goldblum et al. noted that several epithelial abnormalities were seen in achalasia, including high-grade squamous dysplasia, superficial squamous cell carcinoma, and lymphocytic esophagitis [20], exclusively accompanied by diffuse squamous hyperplasia reflecting accelerated cellular turnover and proliferation.

The changes in endocytoscopic images were examined together with the cellular proliferative ability of the mucosal surface in order to evaluate whether the POEM procedure improved cellular proliferation as well as mucosal inflammation. The ultra-high magnification enables us to observe cellular irregularity, similar to conventional pathological images [12,21]. Inoue et al. reported that there was a significant correlation between endocytoscopic images and histopathological images in various gastrointestinal disorders [12,21]. As shown in Figure 5, comparison of endocytoscopic images between before and after POEM revealed significant improvements in the nuclear counts and the irregularity of cellular arrangement. The endocytoscopic nuclear counts after POEM reached almost the same levels as healthy controls. The POEM procedure substantially reduced Ki-67 positivity. There was a significant correlation between Ki-67 expression and endocytoscopic nuclear counts (Figure 4d). Collectively, cellular proliferation ability could be assessed by endocytoscopic images. At the same time, endocytoscopy allows us to obtain information about cellular atypia [12,21].

Fujii et al. reported cases of esophageal squamous cell carcinoma concomitant with achalasia and found higher Ki-67 positivity in cancer lesions than in non-cancerous lesions [22]. Leeuwenburgh evaluated the expressions of both p53 and Ki-67 in achalasia patients and reported that overexpression of p53 could be an early marker of neoplastic progression in achalasia [23]. In esophageal adenocarcinoma, Reid and Kerkhof reported that both p53 and Ki-67 were effective for early discrimination [24,25]. The present data showed decreases of both Ki-67 and p53 expressions after POEM, which implies that POEM could reduce the future risk of developing esophageal squamous cell carcinoma.

Leeuwenburgh documented that Ki-67 expression was not a discriminative factor for carcinogenesis in achalasia patients. However, they targeted the GEJ, which could be easily affected by distention of food and fermentation. Expression of the Ki-67 protein is associated with the clinical course of cell proliferation that is closely linked to tissue inflammation [26]. Leeuwenburgh and his colleagues suggested taking a biopsy from the thoracic esophagus, not from the EGJ, to avoid the effects of severe inflammation. In the present series, biopsies were taken from the middle-thoracic esophagus to avoid sampling severely inflamed mucosa.

Furthermore, advanced achalasia may conceal the dysphagia caused by cancer growth. Stasis of the esophageal contents and a severely inflamed mucosa make it almost impossible to detect early cancers. Improving visualization via the therapeutic intervention allows detailed observation and cancer inspection at an earlier stage. Therefore, we strongly insist that early release of the obstruction is essential for finding early cancers and preventing the development of advanced cancer.

Brossard et al. showed that the incidence of squamous cell carcinoma was eight times higher in untreated patients with achalasia and 4.5 times higher in patients treated with pneumatic dilatation than in those treated with surgical myotomy [27]. Endoscopic myotomy may provide less risk of SCC development with a higher success rate than other options. Endoscopic balloon dilatation has a lower success rate [14] than the POEM procedure. Unlike surgical myotomy, POEM allows us to preserve the anatomical integrity of the LES and possibly minimize postoperative reflux [28,29]. Nevertheless, these LES pressure-lowering therapies could be ultimately complicated by the development of Barrett’s esophagus and adenocarcinoma. Therefore, longer follow-up is obviously necessary to clarify the risk of Barrett’s cancer due to reflux esophagitis, which was observed in some of our cases [19,30].

## Conclusions

POEM could be one of the most effective and less invasive treatment options against dysphagia and reduce the risk of developing esophageal squamous cell carcinoma. Endocytoscopy can be useful for the assessment of esophageal cellular proliferation.

## Competing interests

The authors have nothing to declare.

## Authors' contributions

Guarantor of the article: HM, Specific author contributions: HM, first author of the article treated and followed the patients. HI treated and followed the cohort and composed the database; KO has treated and followed the cohort. KM and NY conducted statistical analysis; YA and FT contributed statistical advice; TN and TH contributed to the pathological examination and analysis. KN and HI, supervisor, devised study protocol and co-wrote the article. All authors read and approve the final manuscript.

## Pre-publication history

The pre-publication history for this paper can be accessed here:

http://www.biomedcentral.com/1471-230X/13/22/prepub
